# The effectiveness of a hybrid mindfulness-based intervention in reducing stress among informal caregivers of people with dementia: a randomized controlled trial

**DOI:** 10.1093/geront/gnaf281

**Published:** 2025-11-23

**Authors:** Patrick Pui Kin Kor, Alex Pak Lik Tsang, Daniel Lok Lam Lai, Justina Yat Wa Liu

**Affiliations:** School of Nursing, The Hong Kong Polytechnic University, Hong Kong, China; School of Nursing, The Hong Kong Polytechnic University, Hong Kong, China; Department of Clinical Psychology, Kwong Wah Hospital, Hospital Authority, Hong Kong, China; School of Nursing, The Hong Kong Polytechnic University, Hong Kong, China

**Keywords:** Caregiver burden, Caregivers, Dementia, Mindfulness-based cognitive therapy, Randomized controlled trial

## Abstract

**Background and Objectives:**

Informal caregivers of people with dementia experience significant caregiving stress. Mindfulness-based interventions are effective approaches for alleviating caregiving stress. However, traditional mindfulness training is intensive and challenging to attend due to the substantial caregiving responsibilities of this population, leading to issues with program attrition and adherence. To address this limitation, a novel Mindfulness-Based Dementia Care Program (MBDCP) was developed by simplifying traditional mindfulness training and utilizing a hybrid delivery model tailored to the needs of dementia caregivers. This study aimed to evaluate the effectiveness of the MBDCP in reducing caregiving stress compared with a brief education program.

**Research Design and Methods:**

A single-blinded, parallel-group randomized controlled trial was conducted with 127 dementia caregivers (MBDCP: *n *= 64; Control: *n *= 63) recruited from three community care centers in Hong Kong. The MBDCP included six weekly 90-min sessions delivered through face-to-face and online formats. Outcomes were assessed at baseline, postintervention, and 6-month follow-up. The primary outcome was perceived stress, whereas secondary outcomes included heart rate variability, mindfulness, depressive symptoms, caregiving burden, dyadic relationships, and neuropsychiatric symptoms of care recipients.

**Results:**

MBDCP significantly improved perceived stress, depressive symptoms, trait mindfulness, and heart rate variability compared with the control group. No significant effects were observed on caregiving burden, dyadic relationships, positive caregiving aspects, or neuropsychiatric symptoms.

**Discussion and Implications:**

The MBDCP effectively reduced stress and improved psychophysiological outcomes in dementia caregivers. These findings highlight the potential of simplified, hybrid mindfulness training to enhance accessibility and effectiveness for this population.

## Pre-registration

The trial was preregistered on ClinicalTrials.gov (NCT05242614).

## Background and objectives

Dementia is characterized by deteriorations in cognitive abilities and functional capacity, often accompanied by behavioral and psychological symptoms of dementia, all of which require significant caregiving. In 2019, over 55 million individuals worldwide are living with dementia, and this figure is expected to increase to 139 million by 2050 (Alzheimer’s Disease International, 2024). In Hong Kong, the caregiving responsibilities for people with dementia commonly fall on informal caregivers. Rooted in the Confucian values of filial piety, adult children are expected to sacrifice their needs for the well-being of their parents; similarly, traditional Chinese marital ethics emphasize duty-bound spousal roles, with spouses frequently providing significant, hands-on support in daily caregiving (Wong et al., 2015). While the intensity and form of caregiving may vary, a strong cultural emphasis on family togetherness generally extends caregiving responsibilities to other relatives, including grandchildren, siblings, and extended kin (Wong et al., 2015). Therefore, Chinese informal caregivers frequently devote more hours to caregiving compared with their Western counterparts (Cai et al., 2025). This can lead to escalating levels of stress as caregivers need to manage a variety of tasks, including assisting with daily activities of living, managing behavioral and psychological symptoms, and balancing work, social roles, and other commitments. The prolonged nature of caregiving for the people with dementia, coupled with uncertainty about disease progression and role conflicts, often leads to significant caregiving stress, which can result in adverse health outcomes, such as high blood pressure and an increased risk of cardiovascular issues (Chattillion et al., 2013). The deterioration of caregivers’ health may in turn impede their ability to continue providing care for people with dementia (Burgdorf et al., 2025). Caregiver role overload in the caregivers is also associated with increased inflammation in care recipients (Tsuker et al., 2025). Despite the high levels of stress associated with dementia caregiving, many caregivers in Hong Kong underutilize supportive services, as they often view caregiving as merely a filial obligation and/or lack sufficient information about the support available to them (Lo et al., 2025). Therefore, reducing stress among informal caregivers is crucial for enhancing their physical and mental well-being and improving the overall quality of care.

Caregiving stress is a dynamic process involving the presence of caregiving stressors and the ways in which caregivers appraise these stressors (Lazarus & Folkman, 1984). Caregiving stressors are unavoidable over the prolonged course of caregiving for people with dementia, as many of these stressors are chronic and inherently difficult to modify (e.g., cognitive decline). Many existing supportive services for caregivers, such as caregiving skills training, focus on strategies to address specific caregiving challenges, but these interventions often yield only temporary stress relief (Dam et al., 2016). An emotion-focused approach, which emphasizes facilitating positive appraisal of these stressors by caregivers, has been advocated as a complementary strategy.

**Figure 1.**
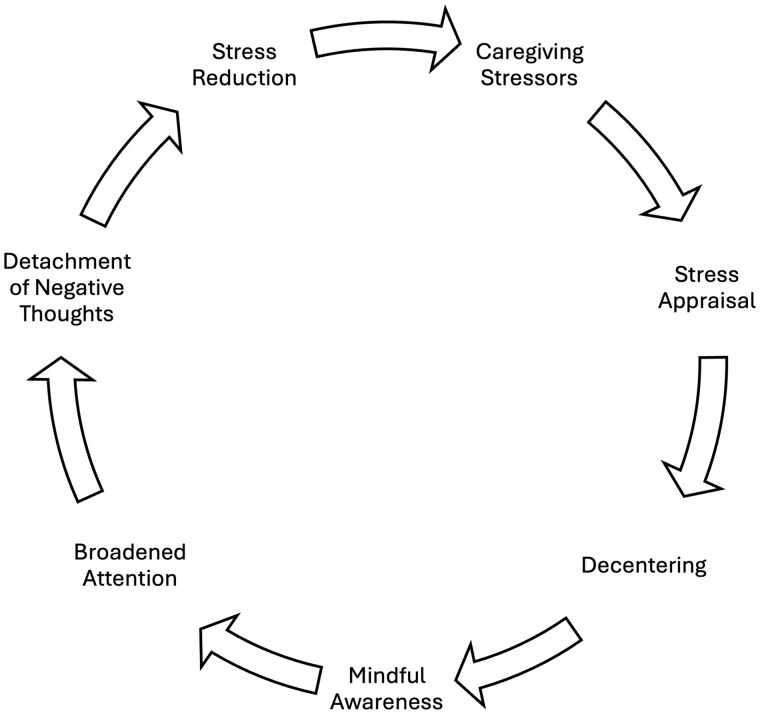
Mindful coping model.

Mindfulness has gained significant traction in Hong Kong as a culturally accepted approach to promoting mental well-being (Ivtzan et al., 2018). Mindfulness-based interventions (MBIs), which focus on fostering present-moment awareness and nonjudgmental attention, offer a promising emotion-focused strategy for supporting caregivers and have been increasingly applied to caregiver populations in Hong Kong. In a recent randomized controlled trial (RCT), Zhang et al. (2025) demonstrated that a brief MBI led to significant improvements in depression, experiential avoidance, family functioning, self-efficacy, and problem-solving coping styles among caregivers of frail older adults. Among dementia informal caregivers, a meta-analysis of 131 RCTs on nonpharmacological interventions found MBIs to have significant and sustainable effects on caregiver mental well-being (Cheng et al., 2020). According to the Mindful Coping Model (Garland et al., 2011), cultivating present-moment self-awareness in a nonjudgmental manner enables caregivers to step outside their negative thoughts through the process of decentering (see Figure 1). By objectively observing these negative thoughts without judgment, caregivers can recognize them as merely constructed realities of the self, from which they can consciously disengage. This shift in perspective allows caregivers to redirect their attention toward new interpretations of negative thoughts, engaging in the process of positive reappraisal. Thus, the processes of decentering and positive reappraisal serve as key mechanisms of stress reduction in MBIs.

Although MBIs have shown potential, evidence regarding their impact on stress reduction for caregivers of people with dementia remains limited. A meta-analysis of the effects of MBIs on caregivers included only five pilot trials, which demonstrated moderate improvements in stress levels in the short term (Kor et al., 2018). However, the long-term benefits of MBIs remain unclear. Most of the reviewed studies employed a standardized MBI format, consisting of weekly sessions lasting 150 min over 8 weeks, along with 45 min of weekly mindfulness practice and a 7.5-hr retreat. This standardized format can be demanding for caregivers, as indicated by high dropout rates of 10.5% to 17.2% (Oken et al., 2010; Whitebird et al., 2013).

Given their caregiving workload, many caregivers struggle to commit to such intensive programs, highlighting the need for a simplified version of MBIs tailored for dementia informal caregivers. It is important to note that a recent narrative review found no association between the total number of in-class hours and the psychological outcomes of participants, suggesting that longer-duration programs do not necessarily translate to greater psychological benefits once participants have mastered mindfulness skills (Carmody & Baer, 2009). Similarly, a three-arm RCT found that both eight-session and four-session MBIs produced comparable improvements in psychological outcomes (Demarzo et al., 2017). These findings suggest that shorter MBIs may be more practical for caregivers with demanding caregiving responsibilities.

In addition to their caregiving roles, informal caregivers are often employed full-time, and work schedule conflicts further complicate their ability to attend face-to-face training programs. As with other psychosocial interventions, MBIs are increasingly being offered in online formats, which can benefit caregivers who find it difficult to participate in face-to-face sessions (Egan et al., 2018; Spijkerman et al., 2016). However, in traditional MBI, the role of the mindfulness instructor extends beyond merely teaching mindfulness skills; they also provide ongoing guidance to help participants effectively learn and practice mindfulness (Spijkerman et al., 2016). Delivering MBIs exclusively online, without adequate guidance, may hinder participants from acquiring and mastering mindfulness skills. For example, a systematic review and meta-analysis reported that the effect size of online MBIs on various psychological outcomes, such as depression and anxiety, was often small (<0.4) when compared with face-to-face MBIs (Spijkerman et al., 2016).

To address limitations of previous MBIs for dementia informal caregivers, this study aims to develop and evaluate the effectiveness of a novel, simplified MBI, the Mindfulness-Based Dementia Care Program (MBDCP), delivered through a hybrid mode. Utilizing a combination of face-to-face and online approaches to deliver MBIs can be a practical solution to improve long-term adherence, sustain mindfulness practices, and realize its intended benefits. An online platform with self-directed learning materials allows caregivers to access the training without disrupting their caregiving responsibilities. On the other hand, face-to-face sessions provide caregivers with opportunities to establish peer support by sharing their experiences and challenges in practicing mindfulness with both peers and the mindfulness facilitator. This hybrid delivery model would be a potential alternative tailored to the unique demands and characteristics of dementia caregivers to help them reduce caregiving stress and improve their overall well-being.

## Research design and methods

**Figure 2.**
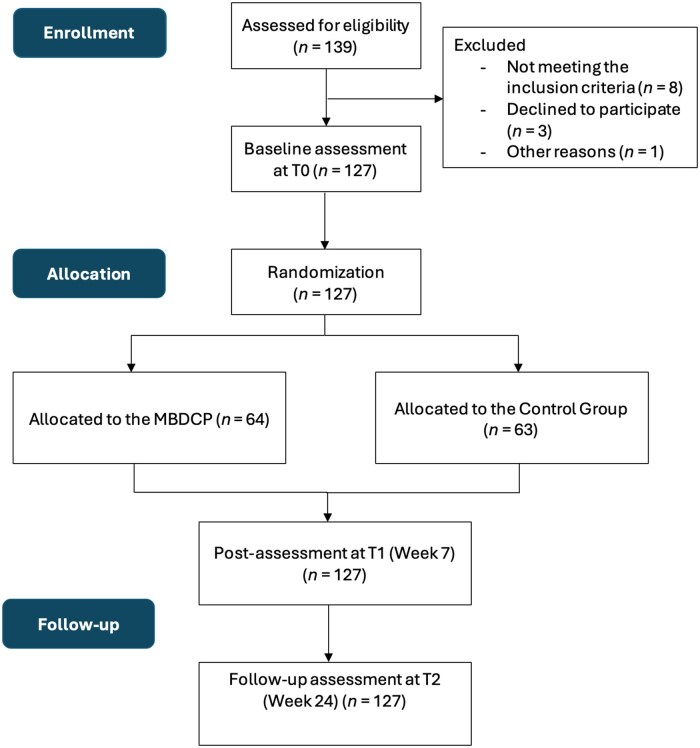
CONSORT flow diagram.

A prospective, single-blinded, parallel-group RCT design was employed. Dementia informal caregivers were randomly assigned to one of two programs: the MBDCP or a brief education program on dementia care (see Figure 2 for the CONSORT flow diagram). The trial was preregistered on ClinicalTrials.gov (NCT05242614). There were no major deviations of the trial protocol. Ethical approval for the study was obtained from the Institutional Review Board of The Hong Kong Polytechnic University (HSEARS20210111006). The trial adhered to the ethical principles of the Declaration of Helsinki (including its subsequent amendments) and the Good Clinical Practice guidelines. The trial was preplanned with no interim analyses or stopping guidelines. There was no participants or public involvement in the design, conduct, reporting, or dissemination of this study. The reporting of this trial conformed to the CONSORT 2025 Statement (see Table S1 in Supplementary Material).

### Participants

The sample size estimation was based on a meta-analysis of MBIs aimed at reducing stress among dementia informal caregivers, which reported an effect size of 0.57 (Kor et al., 2018). Using G*Power 3.1, a sample size of 100 participants would be required to detect this effect with 80% power at a 5% alpha level. To account for an anticipated 20% attrition rate over 6 months, as indicated by findings from our pilot work, a total of 120 participants were targeted.

Dementia informal caregivers were defined as unpaid individuals who have a significant relationship with the people with dementia and are actively involved in providing assistance with activities of daily living. Participants were recruited through convenience sampling, with referrals provided by three local nongovernmental organizations in Hong Kong that offer dementia care services, such as cognitive training and respite care. The inclusion criteria were (1) being a caregiver aged 18 years or older, (2) providing care for an individual with a diagnosis of mild to moderate dementia who resides in the community (based on caregiver’s self-report), and (3) having provided caregiving for at least 6 months prior to recruitment.

The exclusion criteria were (1) prior participation in any structured psychosocial intervention/training or MBIs within 6 months before recruitment; (2) the presence of acute psychiatric or medical conditions that are potentially life-threatening (e.g., suicidal ideation) or that would significantly impair their ability to participate or adhere to the intervention (e.g., acute psychosis); or (3) lack of access to the Internet. Participants were permitted to receive any usual care or support services during the trial. Concomitant care was recorded for baseline comparisons.

### Procedures

Eligible caregivers were screened by a trained research assistant based on predefined inclusion and exclusion criteria. Upon confirming eligibility, written informed consent was obtained from all participants before enrollment. They were informed of their right to decline participation or withdraw from the study at any time without consequences. Randomization was conducted by the research assistant using permuted block randomization with a 1:1 allocation ratio and a block size of four. Caregivers were informed of their group allocation via opaque sealed envelopes distributed on the first day of the intervention. The interventions were structurally equivalent to minimize the likelihood of participants identifying their allocation. The research assistant also served as an independent assessor and was blinded to group allocation. Outcome assessments were conducted in person or via phone interviews. Blinding was achieved by ensuring that the independent assessor was not informed of participants’ group allocation. Potential harms were monitored closely by the research assistant, who notified the research team if any occurred. No harms were observed during the trial.

### Intervention

#### Mindfulness-Based Dementia Care Program

The MBDCP was adapted from the protocol of Mindfulness-Based Cognitive Therapy (MBCT), which has been tested in previous trials aimed at reducing stress among dementia informal caregivers (Kor et al., 2019a; Kor et al., 2019b, 2021). The program was delivered over six consecutive weeks, with each week consisting of a 90-min group-based session using a hybrid approach: The first, second, and sixth sessions were conducted face-to-face, whereas the third, fourth, and fifth sessions were held online. The primary objective of the program was to enhance caregivers’ mindfulness skills through formal and informal mindfulness practices, enabling them to effectively integrate these skills into their daily lives. The face-to-face sessions were conducted in groups of 15 to 18 caregivers and facilitated by a certified mindfulness instructor. These sessions included a variety of mindfulness practices, such as body scanning, mindful walking, and mindful eating, as well as psychoeducation on caregiving and group sharing. The online sessions were designed to facilitate self-directed learning, where caregivers can access tutorial videos on mindfulness through a website. All program content was tailored to address the unique challenges associated with dementia caregiving. Weekly online follow-up sessions, facilitated by the instructor via the website, were conducted to monitor their progress in learning and practicing mindfulness. The online platform also provided caregivers with opportunities to communicate with peers, share their practice experiences, and engage in mutual support.

The face-to-face sessions were audio-taped to ensure intervention fidelity. Using a predefined behavioral checklist, an independent researcher with prior training in mindfulness was invited to listen to the audio recordings and monitor intervention fidelity. The levels of intervention fidelity ranged from 90.2% to 95.3%, with an average of 92.6%. A study protocol of the MBDCP has been published elsewhere (Kor et al., 2022).

#### Control group

The control group participated in a brief education program focused on dementia care and caregiving skills, which included group sharing. This program consisted of six weekly 90-min group sessions and followed a hybrid delivery model, similar to the MBDCP. The delivery format included face-to-face sessions (first, second, and sixth sessions) and online sessions (third, fourth, and fifth sessions). The session frequency, duration, and group size were identical to those of the MBDCP. All sessions were facilitated by a registered nurse with expertise in dementia care or geriatric care.

### Measures

Data collection was conducted at baseline (T0), postintervention in Week 7 (T1), and at the 6-month follow-up in Week 24 (T2).

#### Primary outcome

The primary outcome was perceived stress, which was measured using the Perceived Stress Scale (PSS; Cohen et al., 1983). It consists of 14 items rated on a 5-point Likert scale (0 = *never* to 4 = *almost always*). The total score ranges from 0 to 56, with higher scores indicating greater perceived stress. The Chinese translation of the PSS has been validated in a community sample, with a Cronbach’s alpha of .83 (Huang et al., 2020).

#### Secondary outcomes

Heart rate variability (HRV) was measured as a biomarker of stress using Polar H10 Sensor heart rate monitors. The duration for each time point was 10 min. HRV data were preprocessed using Kubios HRV software. The analysis included both time-domain and frequency-domain indicators. Time-domain measures included mean heart rate (HR), root mean square of successive differences (RMSSD), and standard deviation of NN intervals (SDNN). Frequency-domain measures included low-frequency (LF) power, high-frequency (HF) power, and the low-frequency/high-frequency ratio (LF/HF ratio). The analysis was conducted following the Guidelines for the Standard Measurement and Interpretation of HRV by the European Society of Cardiology and the North American Society of Pacing and Electrophysiology (Task Force of the European Society of Cardiology and the North American Society of Pacing and Electrophysiology, 1996).

Trait mindfulness was assessed using the Five-Facet Mindfulness Questionnaire–Short Form (FFMQ-SF; Baer et al., 2006). It contains 20 items that assess five core domains of mindfulness: observing (awareness of internal and external stimuli, such as sensations, emotions, thoughts, and perceptions), describing (the ability to label internal experiences with words), acting with awareness (attentiveness to present activities while avoiding mindlessness), nonjudging (adopting a nonevaluative attitude toward one’s experiences), and nonreacting (allowing thoughts and feelings to arise and pass without suppression or avoidance). The items are rated on a 5-point Likert scale (1 = *never* to 5 = *very often*), with total scores ranging from 20 to 100. Higher scores reflect greater levels of trait mindfulness. The Chinese version has been validated in the Chinese population, with a Cronbach’s alpha of .83 (Hou et al., 2014).

Depressive symptoms were evaluated using the Center for Epidemiologic Studies Depression (CESD) Scale, which assesses depressive symptoms over a 1-week recall period (Radloff, 1977). The scale consists of 20 items, each rated on a 4-point Likert scale (0 = *less than 1 day* to 3 = *5–7 days*). Total scores range from 0 to 60, with higher scores indicating more severe depressive symptoms. The Chinese version has been validated in the Hong Kong Chinese population, with a Cronbach’s alpha of .86 (Chin et al., 2015).

The Positive Aspects of Caregiving (PAC) Scale was used to measure positive role appraisals of caregivers (Tarlow et al., 2004). It contains nine items that measure two factors: self-affirmation (the confident and capable self-image gained from the caregiving role) and outlook on life (improved interpersonal relationships and a positive life orientation). The items are rated on a 5-point Likert scale (1 = *strongly disagree* to 5 = *strongly agree*). The total score ranges from 9 to 45, with higher scores indicating a more positive caregiving experience. The Chinese version has been validated among Chinese dementia caregivers, with a Cronbach’s alpha of .89 (Lou et al., 2015).

Caregiving burden was measured using the Zarit Burden Interview (ZBI; Zarit et al., 1980). It is a 22-item measure that assesses the impact of caregiving on domains such as physical health, psychological well-being, social life, and economic status. The items are rated on a 5-point Likert scale (0 = *never* to 4 = *nearly always*), with total scores ranging from 0 to 66. Higher scores indicate greater caregiving burden. The Chinese version has been validated among family caregivers, with a Cronbach’s alpha of 0.89 (Ko et al., 2008).

The Dyadic Relationship Scale (DRS) was used to measure dyadic strain (Sebern & Whitlatch, 2007). The scale includes two versions: the care recipient version (DRS-CR, 10 items) and the caregiver version (DRS-CG, 11 items). Each version consists of two subscales: dyadic strain and positive dyadic interaction. Items are rated on a 4-point Likert scale (1 = *strongly disagree* to 4 = *strongly agree*). The subscale for positive dyadic interaction is reverse coded to reflect levels of dyadic strain. The total score ranges from 10 to 40 for the care recipient version and 11 to 44 for the caregiver version. In particular, the DRS-CR has been validated in populations with mild to moderate dementia, demonstrating acceptable reliability, with a Cronbach’s alpha of .70 (Bonds et al., 2021).

The Neuropsychiatric Inventory (NPI) was used to evaluate neuropsychiatric symptoms in people with dementia (Cummings, 2020). It includes 12 symptoms: delusions, hallucinations, agitation/aggression, depression/dysphoria, anxiety, elation/euphoria, apathy/indifference, disinhibition, irritability/lability, and aberrant motor behavior. Each symptom is rated by caregivers on two dimensions—frequency and severity. Frequency is rated on a 4-point Likert scale (1 = *rarely*—*less than once per week* to 4 = *very often*—*once or more per day*). Severity is rated on a 3-point Likert scale (1 = *mild—causing little distress to the patient* to 3 = *severe—very disturbing and difficult to redirect*). The total frequency score ranges from 12 to 48, and the severity score ranges from 12 to 36, with higher scores reflecting greater symptom frequency and severity. The Chinese version has demonstrated a Cronbach’s alpha of .84 (Leung et al., 2001).

#### Sociodemographic, health, and caregiving-related information

Sociodemographic data, including age, gender, education level, and marital status, were collected for both caregivers and people with dementia. For caregivers, additional information on occupational status and income was collected. Health-related data included the presence of chronic illnesses for both caregivers and people with dementia. Caregivers’ self-reported health status was measured using a single-item measure, whereas the functional status of people with dementia was evaluated using the Barthel Index (Mahoney & Barthel, 1965) and the Lawton Instrumental Activities of Daily Living Scale (Lawton & Brody, 1969). Caregiving-related information included whether caregiver resided with the care recipient, their relationship to the care recipient, whether the care recipient was institutionalized, the intensity of caregiving, and whether caregivers were receiving caregiving support.

### Data analyses

Data analyses were conducted using Stata version 18, following an intention-to-treat approach. Descriptive statistics for sociodemographic, health, and caregiving-related information were examined. To address potential confounding, baseline differences between participants in the MBDCP and the control group were assessed using *t* tests for continuous variables and chi-square tests for categorical variables. Descriptive statistics for the study outcomes were also examined. Generalized estimating equations (GEEs) were used to analyze the interaction effects between group (MBDCP vs. control) and time (T0, T1, and T2) on study outcomes. GEE is a marginal modeling approach that utilizes the quasi-likelihood function for estimating population-averaged treatment effects in interventional studies with repeated measures. An exchangeable correlation structure was applied to account for the correlations between repeated measures. The normality of study variables was assessed using the Shapiro–Wilk test to guide the selection of link functions and families for the GEE models (i.e., Gaussian distribution with an identity link for normally distributed variables and gamma distribution with a log link for non-normally distributed variables). Missing data in this study were minimal and primarily came from HRV measurements, ranging from 0.01% to 14.17%. As missing not at random data may bias estimation in GEE models, we performed Little’s MCAR test, which suggested the data were missing completely at random, χ^2^(838) = 838.223, *p *= .491. For significant Group × Time interactions, post hoc pairwise comparisons of the estimated marginal means were conducted to further explore the interaction effects, with Bonferroni corrections for multiple comparisons.

For any covariates showing significant baseline group differences, we conducted sensitivity analyses by including these covariates in the GEE models to adjust for their potential confounding effects. Results from these adjusted models were compared with the primary analyses. All statistical analyses were performed using two-tailed tests, with the significance level set at *p *< .05.

## Results

### Baseline characteristics and descriptive statistics

A total of 127 informal caregivers participated in this study. Of these, 119 participants attended at least 80% of the sessions, resulting in a completion rate of 93.7%. The retention rate was 100%. Table 1 summarizes the baseline sociodemographic, health, and caregiving characteristics. The caregivers had a mean age of 64.278 years (*SD *= 10.766). The majority of caregivers were female (78.7%), had a secondary school education (45.7%), were married (74.0%), unemployed (54.0%), and reported an income of HKD $9,999 or below (37.8%). Caregivers rated their health as moderate (*M *= 3.244, *SD *= 0.663), although 73.2% reported having at least one chronic condition.

**Table 1.** Baseline differences of socio-health-demographic and caregiving-related characteristics.

Variables	Total (*N *= 127)	MBDCP (*n *= 64)	Control group (*n *= 63)	*p*
	*M* (*SD*)/*n* (%)	*M* (*SD*)/*n* (%)	*M* (*SD*)/*n* (%)	
***Caregiver’s characteristics***				
**Age**	64.278 (10.766)	62.609 (10.346)	66.000 (11.001)	.077
**Gender**				
** Male**	27 (21.3%)	19 (29.7%)	8 (12.7%)	<.05
** Female**	100 (78.7%)	45 (70.3%)	55 (87.3%)	
**Education**				
** Primary school or below**	15 (11.8%)	4 (6.2%)	11 (17.5%)	.08
** Secondary school**	58 (45.7%)	34 (53.1%)	24 (38.1%)	
** College or above**	54 (42.5%)	26 (40.6%)	28 (44.4%)	
**Marital status**				
** Never married**	26 (20.5%)	14 (21.9%)	12 (19.0%)	.263
** Married**	94 (74.0%)	49 (76.6%)	45 (71.4%)	
** Divorced/separated**	6 (4.7%)	1 (1.6%)	5 (7.9%)	
** Widowed**	1 (0.8%)	0 (0.0%)	1 (1.6%)	
**Occupation status**				
** Full-time family caregiver**	20 (15.9%)	10 (15.9%)	10 (15.9%)	.478
** Employed**	38 (30.2%)	22 (34.9%)	16 (25.4%)	
** Not employed**	68 (54.0%)	31 (49.2%)	37 (58.7%)	
**Income (in Hong Kong dollars)**				
** $9,999 or below**	48 (37.8%)	22 (34.4%)	26 (41.3%)	.458
** $10,000–19,999**	29 (22.8%)	15 (23.4%)	14 (22.2%)	
** $20,000–29,999**	23 (18.1%)	10 (15.6%)	13 (20.6%)	
** $30,000–39,999**	10 (7.9%)	5 (7.8%)	5 (7.9%)	
** $40,000 or above**	17 (13.4%)	12 (18.8%)	5 (7.9%)	
**Self-reported health**	3.244 (0.663)	3.297 (0.634)	3.190 (0.692)	.368
**Presence of chronic illnesses**				
** No**	34 (26.8%)	19 (29.7%)	15 (23.8%)	.454
** Yes**	93 (73.2%)	45 (70.3%)	48 (76.2%)	
***Care recipient’s characteristics***				
**Age**	83.260 (8.161)	83.938 (8.215)	82.571 (8.114)	.348
**Gender**				
** Male**	41 (32.3%)	18 (28.1%)	23 (36.5%)	.312
** Female**	86 (67.7%)	46 (71.9%)	40 (63.5%)	
**Education**				
** Primary school or below**	69 (54.3%)	36 (56.2%)	33 (52.4%)	.813
** Secondary school**	37 (29.1%)	17 (26.6%)	20 (31.7%)	
** College or above**	21 (16.5%)	11 (17.2%)	10 (15.9%)	
**Marital status**				
** Never married**	2 (1.6%)	0 (0.0%)	2 (3.2%)	.17
** Married**	64 (50.4%)	29 (45.3%)	35 (55.6%)	
** Divorced/separated**	8 (6.3%)	6 (9.4%)	2 (3.2%)	
** Widowed**	53 (41.7%)	29 (45.3%)	24 (38.1%)	
**Presence of chronic illnesses**				
** No**	7 (5.5%)	2 (3.1%)	5 (7.9%)	.235
** Yes**	120 (94.5%)	62 (96.9%)	58 (92.1%)	
**ADL**				
** Total dependency**	14 (11.0%)	12 (18.8%)	2 (3.2%)	.074
** Severe dependency**	29 (22.8%)	15 (23.4%)	14 (22.2%)	
** Moderate dependency**	42 (33.1%)	18 (28.1%)	24 (38.1%)	
** Slight dependency**	23 (18.1%)	11 (17.2%)	12 (19.0%)	
** Total independence**	19 (15.0%)	8 (12.5%)	11 (17.5%)	
**IADL**	2.591 (2.209)	2.328 (2.161)	2.857 (2.242)	.138
***Caregiving information***				
**Reside with care recipients**				
** No**	38 (29.9%)	20 (31.2%)	18 (28.6%)	.742
** Yes**	89 (70.1%)	44 (68.8%)	45 (71.4%)	
**Relationship with caregivers**				
** Spouse**	44 (34.6%)	18 (28.1%)	26 (41.3%)	.07
** Parent/grandparent**	70 (55.1%)	42 (65.6%)	28 (44.4%)	
** Relatives**	11 (8.7%)	4 (6.2%)	7 (11.1%)	
** Friend**	2 (1.6%)	0 (0.0%)	2 (3.2%)	
**Institutionalization of care recipients**				
** No**	125 (98.4%)	62 (96.9%)	63 (100.0%)	.157
** Yes**	2 (1.6%)	2 (3.1%)	0 (0.0%)	
**Average care time per day**				
** 1–4 hr**	43 (33.9%)	19 (29.7%)	24 (38.1%)	.546
** 5–8 hr**	40 (31.5%)	19 (29.7%)	21 (33.3%)	
** 9–12 hr**	5 (3.9%)	3 (4.7%)	2 (3.2%)	
** More than 12 hr**	39 (30.7%)	23 (35.9%)	16 (25.4%)	
**Days spent caregiving per week**	5.315 (2.336)	5.500 (2.204)	5.127 (2.466)	.37
**Receiving caregiving support**				
** No**	64 (50.4%)	33 (51.6%)	31 (49.2%)	.791
** Yes**	63 (49.6%)	31 (48.4%)	32 (50.8%)	

*Note*. *P* values obtained by *t* tests or chi-square tests for continuous outcomes and categorical outcomes, respectively. Self-reported health ranges from 1 (very poor) to 5 (very good). IADL ranges from 0 to 5 for males and 0 to 8 for females. ADL = activities of daily living; IADL = instrumental activities of daily living; MBDCP = Mindfulness-Based Dementia Care Program.

The people with dementia had a mean age of 83.260 years (*SD *= 8.161), with most being female (67.7%). Over half of them had a primary school education or below (54.3%), and the majority were married (50.4%). Chronic illnesses were highly prevalent among people with dementia (94.5%). Most of them demonstrated a moderate level of dependency in activities of daily living (33.1%) and had a mean score of 2.591 (*SD *= 2.209) on instrumental activities of daily living.

Caregiving intensity was high, with 65.3% of caregivers spending more than 5 hr per day providing care, averaging 5.315 days per week (*SD *= 2.336). Over half of the caregivers (50.4%) reported having no source of caregiving support. At baseline, a statistically significant difference in gender distribution was observed between the two groups, with a higher proportion of male caregivers in the MBDCP (*p *< .05). No other statistically significant baseline differences were detected.

Table 2 presents the descriptive statistics of study outcomes across T0 to T2 for participants from the MBDCP and control group. As shown in Table 2, significant deviations from normality were observed in depressive symptoms, positive aspects of caregiving, the severity and distress levels of neuropsychiatric symptoms, and all HRV metrics (*p*s < .01).

**Table 2.** Descriptive statistics of study outcomes.

Variables	MBDCP (*n *= 64)			Control group (*n *= 63)			SW, *p*
	T0	T1	T2	T0	T1	T2	
	*M* (*SD*)	*M* (*SD*)	*M* (*SD*)	*M* (*SD*)	*M* (*SD*)	*M* (*SD*)	
**Perceived stress**	32.234 (3.558)	26.781 (4.891)	27.875 (4.548)	31.889 (3.768)	31.016 (3.003)	29.968 (3.772)	.104
***Secondary outcomes***							
**Mean HR**	71.653 (11.658)	85.362 (19.929)	64.030 (17.708)	68.835 (13.384)	71.463 (17.199)	61.537 (16.835)	<.001
**RMSSD**	38.267 (13.485)	45.989 (18.027)	41.618 (16.456)	37.529 (13.228)	35.637 (13.525)	32.144 (12.584)	<.001
**SDNN**	40.754 (8.628)	44.171 (12.016)	41.453 (10.605)	39.462 (10.695)	37.955 (11.507)	35.879 (11.277)	<.001
**LF power**	302.167 (103.111)	354.486 (138.126)	279.088 (102.738)	378.555 (268.128)	385.270 (357.471)	309.179 (192.254)	<.001
**HF power**	1,744.365 (321.014)	1,915.775 (565.317)	1,872.717 (471.073)	1,603.266 (320.481)	1,344.176 (250.838)	1,329.829 (335.895)	<.001
**LF/HF ratio**	0.176 (0.063)	0.220 (0.133)	0.195 (0.220)	0.241 (0.160)	0.298 (0.246)	0.240 (0.165)	<.001
**Trait mindfulness**	61.406 (8.013)	66.391 (4.796)	67.156 (5.289)	62.968 (8.666)	58.968 (8.179)	58.841 (8.202)	.096
**Depressive symptoms**	38.359 (3.917)	31.063 (4.816)	29.873 (4.874)	39.492 (3.528)	37.190 (3.417)	35.603 (3.129)	<.01
**Positive aspects of caregiving**	14.797 (3.367)	13.781 (2.250)	17.547 (3.911)	14.444 (2.833)	13.111 (2.118)	16.349 (3.012)	<.001
**Caregiving burden**	58.891 (4.738)	58.891 (4.738)	59.734 (5.140)	59.000 (3.976)	59.619 (4.780)	59.254 (4.174)	.073
**Dyadic strain-caregivers**	8.875 (2.043)	12.766 (2.629)	12.312 (2.922)	9.397 (2.083)	12.667 (1.943)	12.651 (1.927)	.471
**Neuropsychiatric symptoms-distress**	27.828 (19.618)	25.219 (5.722)	24.484 (4.595)	29.159 (19.452)	30.302 (8.312)	29.778 (8.208)	<.001
***People with dementia outcomes***							
**Dyadic strain-care recipients**	6.500 (2.182)	6.516 (2.108)	6.516 (2.108)	6.571 (2.069)	6.429 (1.838)	6.016 (1.782)	.289
**Neuropsychiatric symptoms-severity**	17.875 (9.486)	18.641 (3.552)	18.734 (3.734)	19.317 (9.898)	17.302 (3.970)	18.270 (3.629)	<.01

*Note*. HF power = high-frequency power; HR = heart rate; LF power = low-frequency power; MBDCP = Mindfulness-Based Dementia Care Program; RMSSD = root mean square of successive differences; SDNN = standard deviation of normal-to-normal intervals; SW = Shapiro–Wilk test.

### Effects on stress reduction

The main results were shown in Table 3. A statistically significant Group × Time interaction was observed for perceived stress (β = –1.219, *p *< .05). Post hoc pairwise comparisons revealed a reduction in stress in the MBDCP from T0 to T1 (*M_diff_* = –5.453, *p *< .001) and from T0 to T2 (*M_diff_* = –4.359, *p *< .001), whereas no changes were observed in the control group. Stress levels were lower in the MBDCP compared with the control group at T1 (*M_diff_* = –4.235, *p *< .001) and T2 (*M_diff_* = –2.093, *p *< .05).

**Table 3.** Generalized estimating equations results of group and time interaction effect on study outcomes.

Variables	Group (ref.: Control group)			Time (T0–T2)			Group × Time		
	β	*p*	95% CI	β	*p*	95% CI	β	*p*	95% CI
**Perceived stress**	0.445	.696	[–1.789, 2.678]	–0.96	<.05	[–1.708, –0.212]	–1.219	<.05	[–2.273, –0.166]
***Secondary outcomes***									
**Mean HR**	0.091	.146	[–0.032, 0.214]	–0.056	<.01	[–0.094, –0.018]	0.000	.994	[–0.054, 0.053]
**RMSSD**	–0.059	.449	[–0.212, 0.094]	–0.077	<.001	[–0.111, –0.044]	0.119	<.001	[0.072, 0.167]
**SDNN**	–0.002	.971	[–0.120, 0.116]	–0.048	<.01	[–0.078, –0.017]	0.056	<.05	[0.013, 0.099]
**LF power**	–0.263	<.05	[–0.509, –0.017]	–0.099	<.001	[–0.153, –0.044]	0.067	.088	[–0.010, 0.143]
**HF power**	–0.009	.866	[–0.116, 0.098]	–0.095	<.001	[–0.125, –0.065]	0.133	<.001	[0.090, 0.175]
**LF/HF ratio**	–0.363	<.05	[–0.709, –0.017]	0.01	.845	[–0.089, 0.108]	0.046	.518	[–0.094, 0.187]
**Trait mindfulness**	–5.152	<.01	[–8.877, –1.427]	–2.063	<.01	[–3.241, –0.886]	4.938	<.001	[3.280, 6.597]
**Depressive symptoms**	0.02	.559	[–0.046, 0.085]	–0.052	<.001	[–0.074, –0.030]	–0.073	<.001	[–0.104, -0.043]
**Positive aspects of caregiving**	0.002	.979	[–0.111, 0.115]	0.062	<.01	[0.024, 0.100]	0.023	.389	[–0.030, 0.076]
**Caregiving burden**	–0.709	.47	[–2.630, 1.212]	0.127	.594	[–0.340, 0.594]	0.295	.38	[–0.363, 0.953]
**Dyadic strain-caregivers**	–0.437	.491	[–1.682, 0.807]	1.627	<.001	[1.232, 2.022]	0.092	.746	[–0.464, 0.648]
**Neuropsychiatric symptoms-distress**	0.007	.949	[–0.210, 0.224]	0.011	.758	[–0.056, 0.077]	–0.075	.12	[–0.169, 0.019]
***People with dementia outcomes***									
**Dyadic strain-care recipients**	–0.399	.342	[–1.223, 0.424]	–0.278	<.01	[–0.468, –0.088]	0.286	<.05	[0.018, 0.553]
**Neuropsychiatric symptoms-severity**	–0.096	.302	[–0.279, 0.087]	–0.028	.361	[–0.088, 0.032]	0.051	.232	[–0.033, 0.136]

*Note*. HF power = high-frequency power; HR = heart rate; LF power = low-frequency power; ref. = reference group; RMSSD = root mean square of successive differences; SDNN = standard deviation of normal-to-normal intervals.

### Effects on secondary outcomes

Statistically significant improvements were found in time-domain metrics of HRV. A general decrease in mean HR was observed for both groups (β = –0.056, *p *< .01), indicating a trend toward reduced physiological arousal. A statistically significant Group × Time interaction was found for RMSSD (β  =  0.119, *p *< .001). In the MBDCP, RMSSD increased from T0 to T1 (*M_diff_* = 7.723, *p *< .001), reflecting enhanced parasympathetic activity and improved stress resilience, although there was a decrease from T1 to T2 (*M_diff_* = –4.372, *p *< .05). In contrast, the control group showed a decrease in RMSSD from T0 to T2 (*M_diff_* = –5.385, *p *< .001) and from T1 to T2 (*M_diff_* = –3.492, *p *< .05). RMSSD was higher in the MBDCP compared with the control group at T1 (*M_diff_* = 10.353, *p *< .01) and T2 (*M_diff_* = 9.473, *p *< .01), suggesting greater parasympathetic regulation. Similarly, for SDNN, a statistically significant Group × Time interaction was detected (β  =  0.056, *p *< .05). The MBDCP had higher values at T1 (*M_diff_* = 6.215, *p *< .05) and T2 (*M_diff_* = 5.573, *p *< .05), indicating better autonomous nervous system flexibility and stress regulation, whereas the control group exhibited a reduction in SDNN from T0 to T2 (*M_diff_* = –3.583, *p *< .05).

For frequency-domain metrics, a significantly significant Group × Time interaction was found for HF power (β  =  0.133, *p *< .001). In the MBDCP, HF power increased from T0 to T1 (*M_diff_* = 181.394, *p *< .05), suggesting enhanced parasympathetic activity and improved capacity for stress regulation, whereas the control group showed a decrease from T0 to T1 (*M_diff_* = –253.291, *p *< .001) and from T0 to T2 (*M_diff_* = –274.338, *p *< .001). HF power was higher in the MBDCP compared with the control group at T1 (*M_diff_* = 570.481, *p *< .001) and T2 (*M_diff_* = 545.910, *p *< .001). For LF power, both the main effects of group (β = –0.263, *p *< .05) and time (β = –0.099, *p *< .001) were significantly significant, indicating a reduction in LF power over time, with the control group showing higher LF power than the MBDCP, implicating improved autonomic balance and lower autonomic arousal in the MBDCP. There was also an improvement in the LF/HF ratio as indicated by a group effect (β = –0.363, *p *< .05), reflecting greater parasympathetic dominance and more effective stress regulation.

There was an increase in trait mindfulness for the MBDCP, as indicated by a significantly significant Group × Time interaction (β  =  4.938, *p *< .001). In the MBDCP, trait mindfulness increased from T0 to T1 (*M_diff_* = 4.984, *p *< .001) and from T0 to T2 (*M_diff_* = 5.750, *p *< .001), whereas a decrease was observed in the control group from T0 to T1 (*M_diff_* = –4.000, *p* < .05) and from T0 to T2 (*M_diff_* = –4.127, *p *< .01). Dispositional mindfulness levels were higher in the MBDCP compared with the control group at T1 (*M_diff_* = 7.422, *p *< .001) and T2 (*M_diff_* = 8.315, *p *< .001).

Furthermore, a significantly significant Group × Time interaction was found for depressive symptoms (β = –0.073, *p *< .001). While the control group demonstrated a reduction in depressive symptoms from T0 to T2 (*M_diff_* = –3.889, *p *< .001), the MBDCP showed greater improvements over the same period (*M_diff_* = –8.486, *p *< .001). An immediate reduction in depressive symptoms was observed in the MBDCP group at T1 (*M_diff_* = –7.296, *p *< .001). Depressive symptom levels were consistently lower in the MBDCP compared with the control group at T1 (*M_diff_* = –6.127, *p *< .001) and T2 (*M_diff_* = –5.730, *p *< .001).

However, while PAC increased over time for both groups (β  =  0.062, *p *< .01), there was a general increase in dyadic strain over time (β  =  1.627, *p *< .001). The effects of the MBDCP on caregiving burden or the distress levels associated with neuropsychiatric symptoms were statistically nonsignificant.

### Effects on people with dementia

For outcomes related to people with dementia, a significantly significant Group × Time interaction was observed in dyadic strain (β  =  0.286, *p *< .05). However, post hoc tests revealed no significant pairwise comparisons, although a marginal reduction was noted in the control group from T0 to T2 (*M_diff_* = –0.556, *p *= .062). However, the effects on the severity levels of neuropsychiatric symptoms were statistically nonsignificant.

### Sensitivity analyses

Given the baseline differences in gender between participants the MBDCP and control group, a sensitivity analysis was conducted by including gender as a covariate in the generalized estimating equations models. The results remained largely unchanged (see Table S2 in Supplementary Material).

## Discussion and implications

In Hong Kong, the supportive services for caregivers have primarily adopted a problem-focused approach (e.g., respite care) (The Hong Kong Polytechnic University Consulting Team, 2022), highlighting the need for emotion-focused approaches as complementary strategies for achieving long-term stress reduction. MBIs have emerged as effective strategies for alleviating caregiving stress (Kor et al., 2018). However, traditional MBIs are intensive, which can be difficult for caregivers to attend due to their demanding caregiving responsibilities, leading to issues with program attrition and adherence (Kor et al., 2018). To address these limitations, the MBDCP presents a simplified MBI utilizing a hybrid delivery model tailored to the needs of this population. Our findings demonstrate that the MBDCP significantly reduced caregiving stress. Improvements were also observed in trait mindfulness and depressive symptoms. These results indicate that combining face-to-face and online components to deliver MBI is an effective and feasible approach for reducing stress and supporting the mental well-being of informal caregivers of people with dementia.

The improvements in perceived stress specific to the MBDCP align with findings from previous trials of MBI for this population (Brown et al., 2016; Kor et al., 2021; Mallya & Fiocco, 2019). According to the Mindful Coping Model, present-moment, nonjudgmental awareness enables caregivers to disengage from negative thought patterns through decentering, which allows them to view negative thoughts as temporary mental events, fostering more positive appraisals of stress (Garland et al., 2011). This is particularly relevant to dementia informal caregivers, as dementia is an irreversible condition accompanied by stressors such as cognitive decline that are difficult to modify. Given the prolonged trajectory of dementia caregiving, the ability to sustain positive stress appraisals is essential for providing sustainable care. While previous MBI trials have demonstrated its effectiveness for stress reduction, the majority of them have either lacked follow-up assessments (Mallya & Fiocco, 2019; Oken et al., 2010) or employed shorter follow-up periods up to 3 months (Brown et al., 2016). Thus, our findings demonstrate that a hybrid MBI can produce reductions in perceived stress for up to 6 months, offering a sustainable approach to supporting the long-term well-being of dementia caregivers.

The stress-reducing effects of the MBDCP were further corroborated by HRV data. Group-specific improvements in time-domain metrics, such as RMSSD, indicated enhanced parasympathetic regulation, while increases in SDNN reflected overall improvements in autonomic balance. In addition, frequency-domain analyses revealed increased HF power, indicating enhanced parasympathetic activity, along with improved LF power, suggesting more balanced autonomic responses. Few previous trials of MBIs have utilized psychophysiological measures of caregiving stress. Among these, Oken et al. (2010) and Brown et al. (2016) reported null effects of changes in cortisol levels. This lack of effect aligns with research suggesting a nonsignificant association between HRV and cortisol levels. For instance, Looser et al. (2010) found that while cortisol levels were associated with changes in HRV in high-stress circumstances, such a relationship was attenuated during normal or low-stress conditions. This suggests that cortisol may be less sensitive to detecting stressors in lower baseline stress. Similarly, Pulopulos et al. (2018) demonstrated that while a stress-inducing task effectively induced changes in HRV, it did not result in changes in cortisol levels. These findings highlight that HRV could be a more sensitive psychophysiological measure for detecting stress in MBIs. Future studies should consider using multiple psychophysiological indicators, such as HRV and inflammatory responses, to better capture the stress reduction effects of MBIs for informal dementia caregivers.

The MBDCP also led to a significant reduction in depressive symptoms. While both groups demonstrated a decrease in depressive symptoms over time, potentially indicating increased adaptability to caregiving demands, participants from the MBDCP exhibited a more pronounced and immediate reduction at posttest. These findings are consistent with previous trials of MBIs for informal dementia caregivers (Kor et al., 2021; Mallya & Fiocco, 2019), further supporting MBIs as an effective approach to alleviating depressive symptoms in this population. Among caregivers, depressive symptoms are often associated with rumination, the cognitive tendency characterized by the repetitive and excessive analysis of the causes and consequences of negative thoughts, rather than engaging in active problem-solving (Nolen-Hoeksema et al., 2008). Given that the MBCT specifically targets ruminative thoughts, it may be well suited for caregivers who exhibited ruminative tendencies (Cheung et al., 2020). Through sustained mindfulness practice, the MBDCP demonstrated improvements in depressive symptoms maintained for up to 6 months, highlighting its potential to enhance dementia caregivers’ mental well-being over the long term.

Importantly, the MBDCP produced group-specific improvements in trait mindfulness, suggesting that a hybrid delivery model is effective in facilitating mindfulness practices among dementia informal caregivers. Previous trials utilizing the MBCT have reported null effects on trait mindfulness when using a traditional face-to-face delivery mode (Oken et al., 2010). A potential explanation is that six weekly 90-min face-to-face group sessions may be too demanding for caregivers, who must balance their caregiving responsibilities with other life commitments. In Hong Kong, a significant portion of informal caregivers for older adults are employed full-time (The Hong Kong Polytechnic University Consulting Team, 2022). Due to strong filial obligations, these caregivers often devote more hours to caregiving than their Western counterparts (Cai et al., 2025). Indeed, a previous MBCT trial in Hong Kong reported that approximately 20% of participants discontinued their practice, citing a lack of available time as the primary reason (Kor et al., 2021). In MBIs, a key objective is to encourage regular out-of-class practice to integrate mindfulness skills into everyday life. However, if caregivers perceive the intervention as overly intensive, their motivation to engage in home practices may decline, thereby limiting the benefits of MBIs. This highlights the need for simplified and more flexible intervention formats tailored to caregivers’ unique circumstances. For example, Kor et al. (2019b) demonstrated increases in trait mindfulness at both postintervention and 3-month follow-up in a simplified protocol of MBCT for dementia caregivers. Our findings further support the effectiveness of a hybrid approach in addressing these challenges. Reducing face-to-face time and adding online components allowed caregivers to participate flexibly without disrupting their other commitments while still receiving support and guidance from the mindfulness instructor. Importantly, no participants dropped out during the intervention, and the sustained effects observed in our study suggest that participants maintained their mindfulness practice over time. This highlights the potential of hybrid delivery models to enhance accessibility and effectiveness for dementia informal caregivers.

Despite the positive findings on stress reduction and mental well-being, the MBDCP did not demonstrate statistically significant effects on caregiving burden, including increased dyadic strain perceived by caregivers. This result largely aligns with previous studies (Brown et al., 2016; Mallya & Fiocco, 2019). In a meta-analysis of MBIs for dementia informal caregivers, Liu et al. (2018) reported little to no effect on caregiving burden. A potential explanation could be the multidimensional nature of caregiving burden, which encompasses a range of situational and contextual factors and varies among caregivers (e.g., financial strain). In Hong Kong, many caregivers face structural barriers, such as limited access to information, when seeking supportive services like financial subsidies and respite care (The Hong Kong Polytechnic University Consulting Team, 2022). As a result, they continue to face significant stressors that were not directly addressed by the MBDCP, which focused primarily on psychosocial outcomes. In addition, behavioral and psychological symptoms are a major source of caregiving burden (Huang et al., 2012), which were not specifically targeted by the intervention. While mindfulness practices may assist caregivers in responding more constructively to these symptoms (Kor et al., 2021), the short-term nature of the MBDCP may not have provided sufficient time for participants to fully integrate mindfulness skills into their caregiving practices. Given that behavioral and psychological symptoms have a significant negative impact on the quality of the caregiver–care recipient relationship (Huang et al., 2012), the null effects on the severity and distress levels of behavioral and psychological symptoms could also explain the observed increase in caregiver-perceived dyadic strain. This finding is consistent with earlier studies that reported no significant improvements in dyadic relationships following MBIs (Brown et al., 2016). Alternatively, managing behavioral and psychological symptoms and reducing caregiving burden may require a combination of emotion-focused approaches, such as mindfulness, and problem-focused approaches, such as skill training for managing challenging behaviors. Future research should further explore multicomponent interventions that combine mindfulness practices with practical support to address caregiving burden. In addition, the follow-up period in this study was limited to 6 months, which may not have been sufficient to capture long-term changes in caregiving burden. Given the chronic and progressive nature of dementia caregiving, future studies should include longer follow-up periods to evaluate whether MBIs produce cumulative effects on caregiver burden over time.

### Implications for practice

Overall, our findings indicate that the hybrid delivery model of the MBDCP is effective in improving mental well-being among informal caregivers of people with dementia. As it is adapted from the MBCT protocol, this intervention is particularly suitable for caregivers experiencing ruminative thoughts and depressive symptoms (Cheung et al., 2020). Furthermore, as mindfulness is culturally contextualized, our study suggests that a mindfulness-based approach, consistent with other research, is culturally acceptable for stress reduction in this population (Ivtzan et al., 2018). In addition, this approach is well suited for caregivers who are heavily involved in caregiving responsibilities and may find it challenging to attend longer, more intensive interventions or leave their care recipients to participate. By addressing these barriers, the hybrid delivery model enhances accessibility, providing psychosocial support to these vulnerable populations, which is suitable for the fast-paced lifestyle in Hong Kong. The sustained improvements in trait mindfulness observed in our study suggest that participants continued to engage in mindfulness practices even after the program ended. This highlights the potential of the program to support long-term caregiving, which is especially relevant given the long trajectory of dementia, often spanning 8 to 10 years. Given these positive outcomes, the MBDCP offers a nonintensive and flexible delivery format, utilizing online materials that can be easily implemented in community settings. Therefore, more initiatives should focus on integrating this program into standardized supportive dementia care services to benefit the growing number of dementia caregivers. Moreover, due to its nonintensive nature, the MBDCP can also be integrated into other structured interventions, such as caregiving skill-training programs. This underscores its potential as a complementary intervention to further enhance the well-being of informal dementia caregivers.

### Limitations

However, this study has some limitations. First, we recruited only informal caregivers of individuals with mild to moderate dementia, which limits the generalizability of our findings to caregivers supporting individuals with more advanced stages of dementia or those who are institutionalized. Due to practical constraints, dementia staging was based primarily on caregivers’ self-reports of symptom severity. Although caregivers are generally familiar with the symptoms and progression of their care recipients, self-reported data may introduce bias. Future research should incorporate validated dementia staging assessments as part of the inclusion criteria. Second, the use of convenience sampling may have introduced selection bias, suggesting that future studies should aim to recruit samples with more diverse demographic profiles and caregivers across all stages of dementia, including those who are institutionalized. Third, the absence of an MBCT group prevents us from directly evaluating the stress-reduction effects between the MBDCP and traditional MBIs. Fourth, although the Mindful Coping Model suggests that decentering and positive reappraisal are key mechanisms of stress reduction, and there is increasing evidence supporting the effectiveness of MBIs for stress reduction in this population, the present study did not directly measure these proposed mechanisms. Although it is plausible that the MBDCP group experienced reduced stress as a result of increased trait mindfulness, this remains speculative. However, the current sample size did not provide sufficient statistical power to detect mediation effects of the proposed mechanisms. Therefore, future studies are encouraged to use larger sample sizes to further advance our understanding of the mechanisms underlying stress reduction in MBI for this population. Fifth, the lack of cost-effectiveness analyses limits our understanding of the economic feasibility of the MBDCP. Although the hybrid model reduces face-to-face time and lowers interventionist costs, future studies should evaluate its cost-effectiveness to deliver the MBDCP in community settings. We also recommend future research to conduct implementation trials to better understand the scalability and sustainability of the MBDCP. Sixth, caregivers were not involved in the design of the MBDCP due to resource constraints. Although the program was adapted from an established MBCT protocol, the absence of caregiver input may have limited its relevance and responsiveness to their specific needs. Future studies should consider employing a co-design approach to better tailor the program and potentially further enhance its stress reduction effects.

### Conclusion

In conclusion, the MBDCP represents a promising intervention for reducing stress and depressive symptoms among dementia caregivers, with sustained effects observed up to 6 months postintervention. Notably, the program led to significant improvements in trait mindfulness, which were maintained over the follow-up period. These findings suggest that the hybrid delivery model of mindfulness is both effective and flexible, accommodating the demanding caregiving responsibilities faced by informal caregivers of people with dementia. Given the increasing number of dementia caregivers, the MBDCP holds significant potential to enhance caregiver well-being. By addressing the unique challenges faced by this population, such programs can contribute to improving the overall quality of dementia care while supporting the long-term mental health of caregivers.

## Supplementary Material

gnaf281_Supplementary_Data

## Data Availability

The conditions of our ethics approval do not allow us to publicly archive or share the study data with nonmembers of the research team. To seek access to the data, analytic methods, and materials related to this study, interested readers should contact the corresponding author (P.P.K.K.) to discuss data sharing agreement.
